# Structural Re-Alignment in an Immunogenic Surface Region of Ricin A Chain

**DOI:** 10.4137/bbi.s437

**Published:** 2008-02-01

**Authors:** Adam T. Zemla, Carol L. Ecale Zhou

**Affiliations:** Computational Biology for Countermeasures Group, Lawrence Livermore National Laboratory, Livermore, CA, U.S.A. 94550

**Keywords:** protein structure alignment, functional alignment, residue similarity, residue-residue correspondence, alignment method, structure comparison

## Abstract

We compared structure alignments generated by several protein structure comparison programs to determine whether existing methods would satisfactorily align residues at a highly conserved position within an immunogenic loop in ribosome inactivating proteins (RIPs). Using default settings, structure alignments generated by several programs (CE, DaliLite, FATCAT, LGA, MAMMOTH, MATRAS, SHEBA, SSM) failed to align the respective conserved residues, although LGA reported correct residue-residue (R-R) correspondences when the beta-carbon (Cb) position was used as the point of reference in the alignment calculations. Further tests using variable points of reference indicated that points distal from the beta carbon along a vector connecting the alpha and beta carbons yielded rigid structural alignments in which residues known to be highly conserved in RIPs were reported as corresponding residues in structural comparisons between ricin A chain, abrin-A, and other RIPs. Results suggest that approaches to structure alignment employing alternate point representations corresponding to side chain position may yield structure alignments that are more consistent with observed conservation of functional surface residues than do standard alignment programs, which apply uniform criteria for alignment (i.e. alpha carbon (Ca) as point of reference) along the entirety of the peptide chain. We present the results of tests that suggest the utility of allowing user-specified points of reference in generating alternate structural alignments, and we present a web server for automatically generating such alignments: http://as2ts.llnl.gov/AS2TS/LGA/lga_pdblist_plots.html.

## Introduction

Computational methods of protein structure comparison are fundamental to the understanding of protein function and evolution, as well as to applications in medicine and bio-defense. Predictions of potential “druggable” targets on protein surfaces and preferred antigenic regions suitable for diagnostics or therapeutics design, for example, have been derived from computational analyses involving protein structure comparison (Lebeda and Olson, 1999; Olson and Cuff, 1999; Zhou et al. 2005). Success of these endeavors depends on computational accuracy, especially in regions of functional importance or surface regions that serve as ligand binding sites on a protein in the intact, native state. However, structure alignment programs are known to produce differing results, based on the specific approaches and scoring functions that are globally applied (Godzik, 1996; Gerstein and Levitt, 1998). Although superposition of protein structures is frequently ambiguous, and protein structure comparison programs often produce distinct—though perhaps equally valid—results (Godzik, 1996; Zu-Kang and Sippl, 1996), one may wish to obtain a structure alignment and set of residue-residue (R-R) correspondences that match residues in a way that represents an optimal “functional alignment”. Previous studies of ribosome-inhibiting proteins (RIPs), for example, establish an evolutionary conservation among aspartate and glutamate residues in an immunogenic region in close proximity to the active site and suggest that certain glutamate residues may have functional roles associated with protein surface electrostatics (Yan et al. 1997; Lebeda and Olson, 1999). Manual inspection of the structures implies spatial conservation of the residue side chains, yet alignment of these residues is problematic using standard structure-based computational methods. The failure of standard methods to “correctly” align a set of residues known to be highly conserved among these structure-function homologs prompted us to re-examine protein structure comparison and alignment criteria in non-structure core regions.

Protein structure comparison programs typically use uniform parameters (e.g. alpha-carbon (Ca) positions) along the entire peptide chain and apply them to all residues (Holm and Sander, 1993; Zu-Kang and Sippl, 1996; Shindyalov and Bourne, 1998; Chiang et al. 2003; Kawabata, 2003; Zemla, 2003; Krissenel and Henrick, 2004). Such approaches have proven to be reasonably successful in aligning proteins and generating R-R correspondences. Correct and accurate alignment of hydrophobic packed structure core regions or regions composed of secondary structure elements is the implied goal of these methods. However, functional sites usually comprise (or at least include) surface residues within less highly structured regions. Furthermore, structural and chemical (side-chain) differences in functional regions are expected to ultimately be responsible for observed differences in function, such as host-range changes based on differential binding to species-specific cell surface receptors or kinetic and substrate differences between related enzymes. Therefore, when comparing two related proteins, correct “functional” alignment and R-R correspondence is important for predicting differential function or binding site characteristics and for applications in computational design of reagents for diagnostics and therapeutics that are specific for the proteins of interest.

In studying the A chain of ricin we determined that criteria for comparison of structurally significant regions may not necessarily yield satisfactory R-R correspondences among residues lying in functional regions (Zhou et al. 2005); computations based on strict correspondence between locations of alpha carbons do not always correctly determine R-R correspondences for these residues. An examination of the literature reveals an awareness of this failing of structure comparison programs (Gerstein and Levitt, 1998; Lackner et al. 2000), but reveals no method that adequately solves the problem. The ProSup software performs a Ca-based alignment and then applies a post-analysis filter using a beta carbon (Cb) alignment to flag R-R assignments that may deserve further inspection. However, no attempt is made to determine whether a “suspect” R-R correspondence may indeed be incorrect, or to determine an improved method for making a correct alignment based on a truly representative point of comparison (e.g. perhaps neither Ca nor Cb) or on side-chain characteristics, or by applying different methods to different parts of the structure, such as structure core regions vs. loops.

We compared alignments generated by several sequence and structure alignment programs to determine whether existing methods would align residues at a highly conserved position within RIPs (Lebeda and Olson, 1999). Here we propose an alternate approach to standard Ca-based methods, which for a known immunogenic surface loop region of the ricin A chain aligns a highly conserved aspartate residue with its counterparts (aspartate alternating usually with glutamate, and occasionally with aspargine or glutamine) in other RIPs, yielding an alignment that is consistent with conservation of residue position as well as residue similarity. We present the results of tests whereby we generated a series of structure alignments based on varying points of reference upon which the alignment calculations were based, and we present a web server, which allows the user to input sets of protein structure segments and to specify a point in space to be used for structure-based alignment calculations.

## Methods

### Sequence and structure alignments

**Figure 1:**
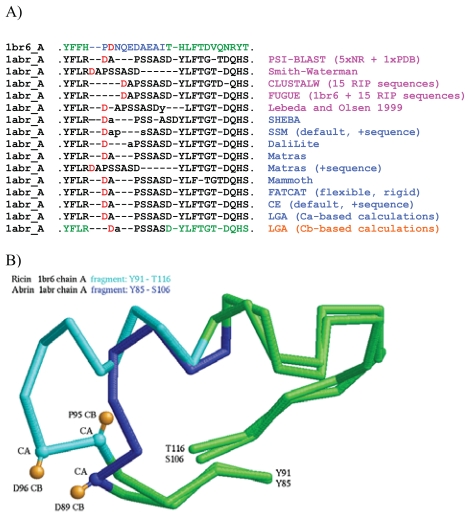
Sequence and structure alignments between ricin (1br6_A; Y91-T116) and abrin (1abr_A; Y85-S106). A) Summary of residue-residue (R-R) correspondences in a surface region containing a conserved aspartate (red). Column at right indicates programs and settings used to generate the correspondences (pink: sequence alignment programs, blue: structure alignment programs, orange: structure alignment calculated on Cb using LGA). Lower-case letters indicate residues that were not assigned correspondence, due to distance cutoffs being exceeded. For CLUSTALW and FUGUE calculations we used the sequences of 15 RIPs listed in Fig. 4. B) Detail of structural alignment between 1br6_A and 1abr_A generated using LGA on the beta-carbon setting. Orange: beta carbons of D96 (1br6_A), P95 (1br6_A) and D89 (1abr_A). A, B) Green: residues that produced consistent R-R correspondences regardless of alignment method used. Light or dark blue: residues that produced inconsistent R-R correspondences.

R-R correspondences derived from global alignments between the structures of abrin-A (1abr_A) and ricin A chain (1br6_A), generated by four sequence alignment programs (PSI-BLAST (Altschul et al. 1997) with five iterations on the NR non-redundant sequence database + a final iteration on PDB), Smith-Waterman (Smith and Waterman, 1981), CLUSTALW (Thompson et al. 1994)), FUGUE (Shi et al. 2001) and by eight structure comparison programs (CE (Shindyalov and Bourne, 1998); DaliLite (Holm and Park, 2000); FATCAT (Ye and Godzik, 2004); LGA (Zemla, 2003); MAMMOTH (Olmea et al. 2002); MATRAS (Kawabata, 2003); SSM (Krissenel and Henrick, 2004); SHEBA (Jung and Lee, 2000)) were compared to determine whether the highly conserved aspartate residues (D89/D96) would be assigned corresponding positions by any of the alignment programs (Fig. 1).

### Comparison of fragments of structure alignments

**Figure 2:**
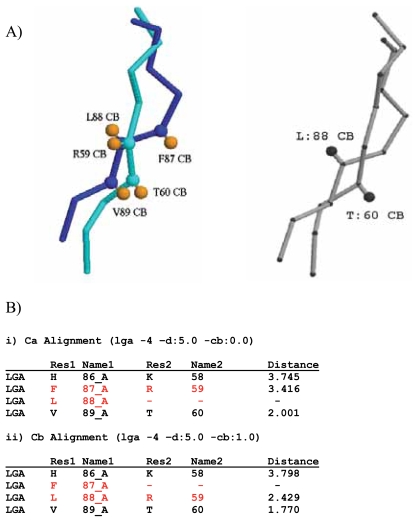
Comparison of fragments of structure alignments calculated for human phosphatidylethanolamine protein (1bd9; E83-N91) and the calcium-phospholipid binding domain from cytosolic phospholipase A2 (1rlw; R55-F63) by LGA and ProSup. A) Left: alignment calculated by LGA. Light blue: cytosolic phospholipase A2. Dark blue: human phosphatidylethanolamine protein. Orange: beta carbons of residues aligned using LGA beta-carbon setting (L88 with R59, V89 with T60). Right: alignment calculated by ProSup (adapted from Lackner et al. 2000. B) Output from LGA comparisons of 1bd9 (Res1) and 1rlw (Res2) using standard alpha-carbon (i) and beta-carbon (ii) alignments. Red: residues whose R-R correspondences differ between (i) and (ii).

Global structure alignments of 1bd9 and 1rlw were performed using LGA on the Ca and Cb settings, and the resulting structure alignments were compared to those obtained using ProSup (Lackner et al. 2000). R-R correspondences and distance calculations were extracted from each structure alignment (Fig. 2).

### Determination of structural deviations between residues in pairwise structure alignments

**Figure 3:**
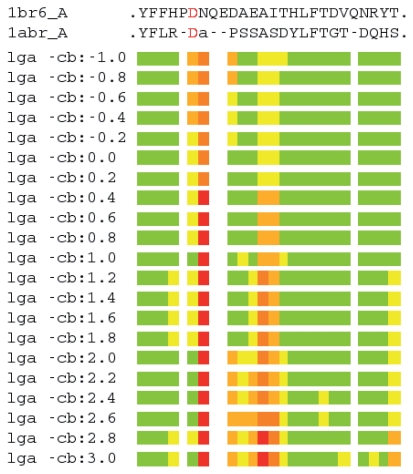
Graphical representation of structural deviations between residues of 1br6_A (ricin) and 1abr_A (abrin) aligned using LGA with varying points of representation. Each bar represents a different superposition, using a distinct point of representation. Shown are structural deviations for the alignment shown in the first and last sequence fragments of Fig. 1A. Colored bars indicate R-R distance ranges: residues superimposed below 2.0 Ǻ are in green, below 4.0Ǻ are in yellow, below 6.0Ǻ are in orange, below 8.0Ǻ are in brown, and at or above 8.0Ǻ are in red. Lower-case letter indicates residue that was not assigned correspondence, due to distance cutoffs being exceeded.

LGA was used in sequence-dependent mode to superimpose ricin A chain (1br6_A) and abrin-A (1abr_A) (Zemla, 2003). Sequence-dependent mode imposes a fixed R-R correspondence when calculating an optimal alignment. A series of superpositions was generated; each superposition was calculated using a different input “-cb” parameter value, which specifies a point of representation along a vector in the direction from the alpha carbon to the beta carbon, with the point of origin being 0.0 at the alpha carbon (-cb:0.0). Possible points of representation range from below the alpha carbon (negative values) to beyond the beta carbon (values >1.0). Points of representation were selected spanning from −1.0 to 3.0 in increments of 0.2, where 1.0 unit corresponds to Cb-Ca distance between the alpha carbon and beta carbon atoms (Fig. 3).

### Pairwise global structure alignments with varying points of representation

**Figure 4:**
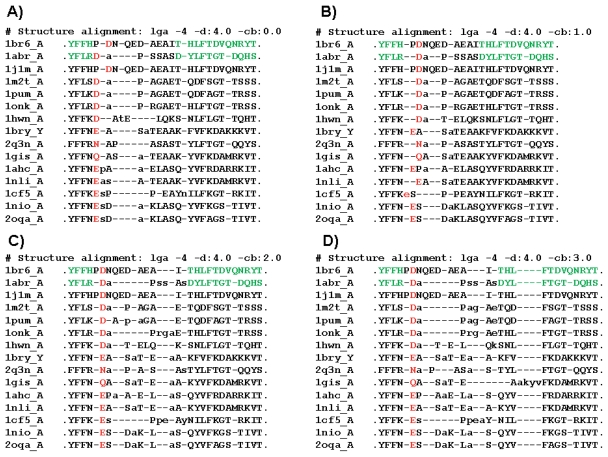
Fragments of pairwise LGA structure alignments, using varying points of representation, between ricin A chain (1br6_A; Y91-T116) and selected RIPs from PDB. Shown are fragments (corresponding to 1br6_A Y91-T116) of 4 representative alignments. Red: Conserved D/E/N/Q residues. Green: Residues that produced consistent R-R correspondences regardless of alignment method used (see Fig. 1). Lower-case letters indicate residues that were not assigned correspondence, due to distance cutoffs being exceeded. A) LGA –cb:0.0. B) LGA –cb:1.0. C) LGA –cb:2.0. D) LGA –cb:3.0.

Pairwise global structural alignments were generated for 15 RIPs selected from PDB using LGA with varying points of representation along a Cb-Ca vector, ranging from 0*Cb (the Ca position; -cb:0.0) to 3*Cb position (-cb:3.0). RIPs were selected from more than 50 PDB structures based on sequence diversity and non-redundancy within the region of interest (corresponding to residues Y91-T116 of 1br6_A). 1j1m_A, which had been solved at very high resolution (1.5 Ǻ), was included as a control for the alignments with the target structure, ricin (1br6_A) (Fig. 4).

## Results

### Beta-carbon alignment

Structure alignment of ricin with other plant and bacterial lectins is known to be problematic in an immunogenic surface region (Lebeda and Olson, 1999; Olson et al. 2004). This region contains a highly conserved residue (aspartate (D) in ricin and abrin), possibly involved in rRNA substrate binding and catalysis (Huang et al. 1995; Olson, 1997; Olson and Cuff, 1999). This residue has been hypothesized to alternate as glutamate (E) or aspartate (D) in ribosome-inhibiting proteins (RIPs) (Lebeda and Olson, 1999), yet a pair-wise structural alignment between ricin (PDB entry 1br6_A) and at least 32 other RIPs demonstrated that the conserved residue (D96) in ricin does not align with the functionally corresponding residue in each of the other proteins when using a standard Ca-based structure comparison method (Zemla, 2003; Zhou et al. 2005). We tested a variety of sequence (PSI-BLAST (Altschul et al. 1997); Smith-Waterman (Smith and Waterman, 1981); CLUSTALW (Thompson et al. 1994); FUGUE (Shi et al. 2001)) and structure (Chiang et al. 2003; CE (Shindyalov and Bourne, 1998), DaliLite (Holm and Park, 2000); FATCAT (Ye and Godzik, 2004); LGA (Zemla, 2003); MAMMOTH (Olmea et al. 2002); MATRAS (Kawabata, 2003); SSM (Krissenel and Henrick, 2004); SHEBA (Jung and Lee, 2000)) alignment programs to determine whether any of them would align the corresponding aspartate residues of ricin A chain and abrin-A (Fig. 1): most of the programs aligned aspartate D89 (1abr_A) with proline P95 (1br6_A), and none aligned the corresponding aspartate residues. Ca-based structure alignments yielded unsatisfactory juxtaposition of the D residues, even when using “sequence information mode” as provided in the SSM, MATRAS and CE programs (Fig. 1A). Close examination of the alignments of ricin A chain with 32 RIPs (Zhou et al. 2005) suggests that shifting a single residue to the right within the RIP sequences would align residue D96 of ricin with aspartate or glutamate residues of the structural homologs. Although such a shift would not be justified based on Ca structural data, a test performed using the LGA program (Zemla, 2003) with Cb atoms as points of reference among structures yielded correspondence of D96 (1br6_A) and D89 (1abr_A) (last alignment of Fig. 1A). Visual inspection of this Cb alignment revealed that the beta carbon positions in P95 (1br6_A) and D89 (1abr_A) pointed in opposite directions, whereas the beta carbons of D96 (1br6_A) and D89 (1abr_A) were closer than were their respective alpha carbons, and were pitched in approximately the same direction (Fig. 1B). The difficulty in automated detection of this R-R correspondence is due in part to insertions (extra residues) in 1br6_A relative to 1abr_A. However, as indicated in Fig. 1, displacement of the alpha carbon of D96 relative to that of D89 is compensated by means of conservation of the spatial placement of the side chains. The success of the Cb alignment in matching D96 (1br6_A) with D89 (1abr_A) is explained by the beta carbon’s proximity to side-chain atoms.

The D/E/N/Q mis-alignment within the RIP family of proteins is representative of a more general limitation of structure alignment programs. We also revisited a structural alignment performed using the ProSup program to determine whether using LGA with a point of representation at the beta carbon would confirm a putative mis-alignment detected using the beta-carbon post-analysis filter of Lackner et al. (2000). Fig. 2 illustrates a putative mis-alignment between regions of human phosphatidylethanolamine protein (1bd9) and the calciumphospholipid binding domain from cytosolic phospholipase A2 (1rlw). ProSup’s Cb post-analysis filter flags a putative mis-alignment at positions corresponding to L88 of 1bd9 and T60 of 1rlw (Fig. 2A). Structure comparisons using LGA on default (Ca) (Fig. 2Bi) vs. Cb (Fig. 2Bii and 2A, left) settings yield alternate R-R correspondences. The Cb analysis using LGA shifted residue R59 of 1rwl from a R-R correspondence match with F87 of 1bd9 to L88, representing a R-R correspondence that is more satisfactory based on orientations of the respective residue side chains (Fig. 2A).

### Alternate point representations

In order to examine the effects of using alternate points of representation (in addition to the alpha and beta carbons) in aligning protein structures, we modified our local-global alignment (LGA) software (Zemla, 2003; Zemla et al. 2005) to accept an input parameter that would adjust the structure coordinates of each protein to represent each residue by a designated point along a line connecting the alpha and beta carbons. We then revisited the structural alignment of abrin-A (1abr_A) and ricin A chain (1br6_A), focusing our attention on the region in the vicinity of residue D96. By sliding the point incrementally, we generated a set of alignments and observed a subset of point representations that yielded a small (less than 2 Ǻ) distance deviation between D96 (1br6_A) and D89 (1abr_A) occurring at 1.0*Cb (Cb position -cb:1.0), from 2.0*Cb to 2.6*Cb, and at 3.0*Cb (distances from the alpha carbon) (Fig. 3). We performed these alignment calculations in “sequence-dependent mode”, wherein the LGA program calculates optimal superposition based on a fixed R-R correspondence, in this case corresponding to that obtained using LGA on the Cb setting (see Fig. 1). This test demonstrates that residues on either side of the loop region (YFFH and THLFTDVQNRY in 1br6_A) are tightly aligned (in most cases less than 2 Ǻ distance deviation) between 1br6_A and 1abr_A regardless of the point of representation used in the alignment. This observation speaks to the stability of the alignment and to the confidence with which one can assert the R-R correspondences. Furthermore, this observation is consistent with that of Figure 1, in which alignment results using several sequence and structure alignment programs produced very similar R-R correspondences for these residues. This test also demonstrates that assignment of R-R correspondences between residues within a loop region can be difficult. Whereas several of the corresponding residues (D96-D89, D100-P91, A101-S92, E102-S93) have small distance deviations (under 4Ǻ) for most points of representation, others (N97-A90, A103-A94, I104-S95) have rather large deviations (greater than 4 Ǻ) for most. Correspondence between N97 and A90, for example, cannot likely be justified using any point of representation along a vector connecting the alpha and beta carbons, nor can Q99 or E100 of 1br6_A be assigned correspondence to any residue of 1abr_A with any degree of confidence.

Additional tests using varying points of representation to align 15 selected RIP structures taken from the PDB were performed to determine how well alternate points of representation faired in aligning the highly conserved D/E/N/Q residues (Fig. 4). When a point of representation corresponding to the alpha carbon was used (Fig. 4A), only the aspartate in 1j1m_A (a ricin Achain) was assigned correspondence to D96 of 1br6_A (ricin). Moving the point of representation to the beta carbon (Fig. 4B) resulted in correspondences being assigned between D96 of 1br6_A and the corresponding conserved residue in 9 of 14 RIPs. With respect to R-R correspondence between conserved D/E/N/Q residues, unanimity was achieved only when the point of representation had been moved to 2*Cb (Fig. 4C), and was maintained at 3*Cb (Fig. 4D). It should be noted that residues in 1br6_A and 1abr_A that were observed to have “stable” R-R correspondences regardless of the program used (Fig. 1A) and regardless of the point of representation used (i.e. by LGA; Fig. 3) to generate the alignment displayed consistency in terms of R-R correspondence (green residues marked in 1br6_A and 1abr_A sequences and all corresponding residues in Fig. 4), with the exception of four residues from 1gis_A (akyv), for which R-R correspondences differed in the 3*Cb alignment (Fig. 4D) due to a shift in the alignment. However, little consistency in R-R correspondence was observed within the loop region (PDNQE-DAEAI in 1br6_A) when sliding the point of representation incrementally from 0.0*Cb through 3.0*Cb (Fig 4), with the exception of the highly conserved D/E/N/Q residues which align at “-cb:” values of 2.0 (2.0*Cb) or higher.

## Discussion

Although the above examples clearly indicate that a Ca-based mis-alignment with respect to chemical (residue) and spatial conservation can be detected and “corrected” by manual inspection of locally applied Cb analysis, this process is by no means simple in the general case. Whereas ProSup’s filter detected the mis-alignment illustrated in Fig. 2, it also detected 18 other potential mis-alignments out of 74 residues aligned using its standard method, implying either that the Ca method was unsuccessful in determining correct alignment over as much as 24% of the protein, or that the filter had a rather high false positive rate. In either case, it is clear that alternate criteria for alignment may be called for depending on whether a method is being applied to regions defining the structural core of a protein vs. regions elsewhere, for example. In order to reverse an alignment acquired using a standard, uniformly applied method, one must 1) determine when it is appropriate to apply alternate criteria for structure alignment, 2) have a meaningful metric (i.e. scoring function) that can determine when the alignment correction should be applied, and 3) incorporate into the metric information about chemistry in the local context in order to determine whether the alignment is consistent with respect to the chemical characteristics of the residues being aligned. These requirements must be met in order to re-align, with confidence, regions of proteins, or re-assign R-R correspondences between residues that in some cases may be presumed significant in terms of biological function. We propose that current approaches to structure alignment stand to be improved by applying differential analyses along the protein chain depending on local structure context, for instance by exploring environmental profiles as discussed in other papers (Jung and Lee, 2000; Shi et al. 2001). Specifically, Ca-based alignments likely work well within structure core regions, whereas methods that incorporate residue position might prove helpful in determining satisfactory residue-residue correspondences in non-core regions, such as surface loops.

It is interesting to note that the variability-based sequence alignment method devised by Fygenson et al. (2004) comes close to structure-based alignment methods for comparison of the closely related alpha and beta tubulins, recapitulating a correlation between functional residue conservation and structural conservation. This and various other methods that have been devised to identify functional residues in proteins, such as residue interaction graphs (Amitai et al. 2004) or statistical methods reviewed by Ahola et al. (2004) could reasonably be used in conjunction with structure-based alignment methods to determine reasonable alignments based on functional considerations.

At http://as2ts.llnl.gov/AS2TS/LGA/lga_pdblist_plots.html we provide a service whereby the user may generate alternate alignments based on defined points of comparison representing residue positions along the peptide chain. This service is intended to enable study of specific cases in which sequence- or structure-based alignments using standard methods are suspect in functional regions. It should be stressed that applying alternate points of representation across the entirely of the protein chain in our test producing Fig. 3 did not always produce acceptable alignments with respect to other portions of the proteins (data not shown). We therefore do not advocate applying alternate point representations blindly among a set of protein structures, but offer that the ability to control the point of reference by which structures are compared provides the researcher with an additional analytical tool for investigating alternate structural alignments biased toward residue (or other) positions when such an approach is warranted either by existing information about residue conservation or as a method of scanning peptide chains for possible occurrences of unexpected R-R spatial correspondence.
